# Supplementary material for: A neurovascular high-frequency optical coherence tomography system enables in situ cerebrovascular volumetric microscopy
Source: Nat Commun. 2020 Jul 31;11:3851. doi: 10.1038/s41467-020-17702-7 (PMC7395105; doi:10.1038/s41467-020-17702-7)
Supplement: Supplementary file 1 — Supplementary Information [file 41467_2020_17702_MOESM1_ESM.pdf]

Supplementary Information

A high-frequency optical coherence tomography endovascular probe enables in situ cerebrovascular volumetric microscopy

Ughi, G. J. et al.

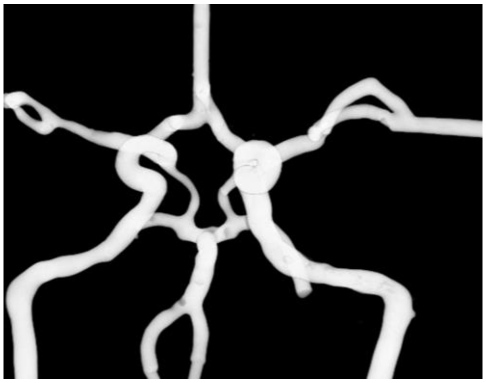

Supplementary Fig. 1. **Bench model of the full, patient-specific circle of Willis**. Merging of the divisions of the middle cerebral and anterior cerebral arteries bilaterally was performed to reduce the complexity of inputs and output of the flow circuit. For the same purpose, individual branch arteries such as the ophthalmic and superior cerebellar arteries were removed.

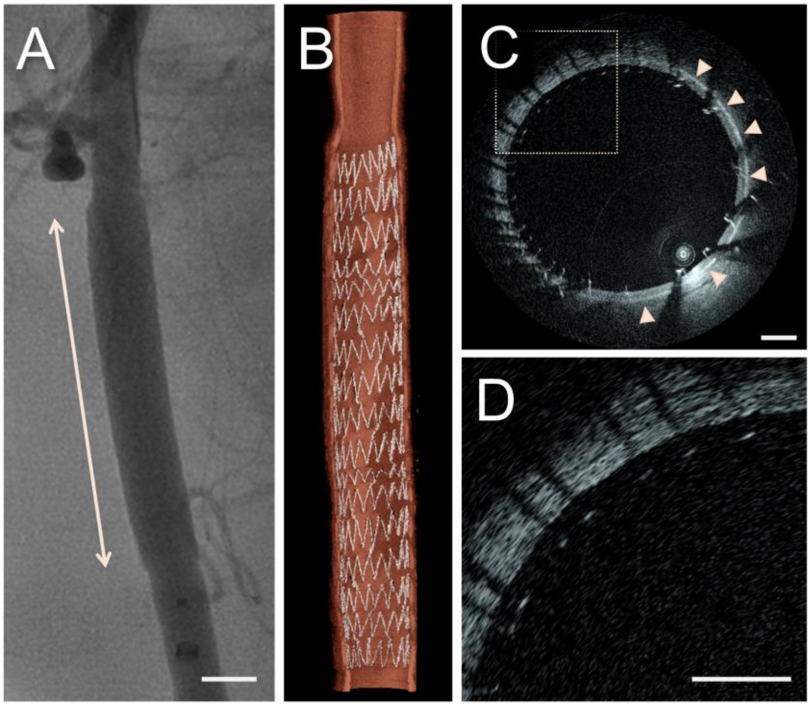

Supplementary Fig. 2. **Imaging of arteries > 5 mm in diameter**. (**A**) imaging was performed in a swine common carotid artery with a maximum diameter of approximately 5.9 mm following the deployment of a Precise Pro Rx Carotid Stent System (Cordis); the stented segment is indicated by the arrow. (**B**) HF-OCT three-dimensional rendering. (**C**) Cross sectional imaging illustrating the extended field-of-view of HF-OCT. Even when the imaging catheter is located in an eccentric position within the arterial lumen, the entire stent and the arterial wall are visualized with sufficient brightness and illumination (**D**). The arrowheads indicate the external elastic membrane (EEL). Scale bars are equal to 1.0 mm. Imaging was repeated in a total of n = 5 common carotid arteries form n = 5 animals following the deployment of a carotid stent system.

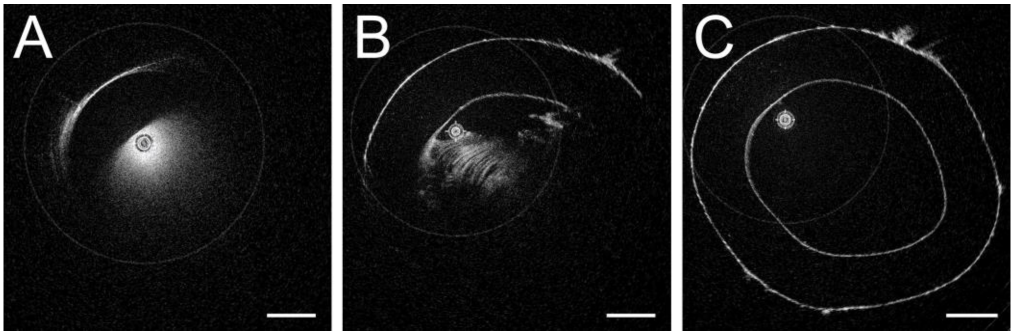

Supplementary Fig. 3. **Blood clearance characterization in a patient-specific vascular model of the circle of Willis**. HF-OCT images were classified into three different categories: (**A**) blood obscuring the field of view, (**B**) partial clearance, (**C**) complete clearance. In all cases, the contrast media injection rate was increased from a value of 1 ml/sec, using increments of 0.5 ml/sec, until a complete clearance state was detected. Scale bars are equal to 1 mm. The experiment was repeated in this model in n = 4 different anatomical locations, at the level of the internal carotid artery, middle cerebral artery, vertebral artery, and basilar artery.
