# Supplementary material for: A neurovascular high-frequency optical coherence tomography system enables in situ cerebrovascular volumetric microscopy
Source: Nat Commun. 2020 Jul 31;11:3851. doi: 10.1038/s41467-020-17702-7 (PMC7395105; doi:10.1038/s41467-020-17702-7)
Supplement: Supplementary file 3 — Reporting Summary [file 41467_2020_17702_MOESM3_ESM.pdf]

nature research

Corresponding author(s): Matthew J Gounis

Last updated by author(s): Jul 1, 2020

Reporting Summary

Nature Research wishes to improve the reproducibility of the work that we publish. This form provides structure for consistency and transparency in reporting. For further information on Nature Research policies, see Authors & Referees and the Editorial Policy Checklist.

Statistics

For all statistical analyses, confirm that the following items are present in the figure legend, table legend, main text, or Methods section.

n/a	Confirmed	
☐	☒	The exact sample size (*n*) for each experimental group/condition, given as a discrete number and unit of measurement
☒	☐	A statement on whether measurements were taken from distinct samples or whether the same sample was measured repeatedly
☐	☒	The statistical test(s) used AND whether they are one- or two-sided<br>*Only common tests should be described solely by name; describe more complex techniques in the Methods section.*
☒	☐	A description of all covariates tested
☒	☐	A description of any assumptions or corrections, such as tests of normality and adjustment for multiple comparisons
☒	☐	A full description of the statistical parameters including central tendency (e.g. means) or other basic estimates (e.g. regression coefficient) AND variation (e.g. standard deviation) or associated estimates of uncertainty (e.g. confidence intervals)
☒	☐	For null hypothesis testing, the test statistic (e.g. *F*, *t*, *r*) with confidence intervals, effect sizes, degrees of freedom and *P* value noted<br>*Give P values as exact values whenever suitable.*
☒	☐	For Bayesian analysis, information on the choice of priors and Markov chain Monte Carlo settings
☒	☐	For hierarchical and complex designs, identification of the appropriate level for tests and full reporting of outcomes
☒	☐	Estimates of effect sizes (e.g. Cohen's *d*, Pearson's *r*), indicating how they were calculated

Our web collection on statistics for biologists contains articles on many of the points above.

Software and code

Policy information about availability of computer code

Data collection	DSA and VasoCT images were generated using Allura FD20 and XtraVision, respectively (Philips Medical Systems, Best, the Netherlands). HF-OCT image were generated using Gentuity imaging console (Sudbury, MA).
Data analysis	Raw statistics (mean, standard deviation) were calculated using Microsoft Excel Version 1905. Fleiss's kappa for inter-rater agreement was calculated using R Version 3 (Vienna, Austria). Manual image analysis was performed in ImageJ Version 1.51 (NIH, Bethesda, MD) and 3D renderings were manually generated using Osirix MD V.10 (Pixmeo, Geneva, Switzerland), both open source programs. As such, no custom code/software code was used to process data obtained from the imaging consoles. Only manual processing of data to generate 3D images was applied and fully described in the Materials and Methods section of the paper (Three-dimensional HF-OCT renderings paragraph). All used software is reported on the manuscript.

For manuscripts utilizing custom algorithms or software that are central to the research but not yet described in published literature, software must be made available to editors/reviewers. We strongly encourage code deposition in a community repository (e.g. GitHub). See the Nature Research guidelines for submitting code & software for further information.

Data

Policy information about availability of data

All manuscripts must include a data availability statement. This statement should provide the following information, where applicable:

- Accession codes, unique identifiers, or web links for publicly available datasets
- A list of figures that have associated raw data
- A description of any restrictions on data availability

The imaging data that support the findings of this study are available from the corresponding author upon reasonable request.

Field-specific reporting

Please select the one below that is the best fit for your research. If you are not sure, read the appropriate sections before making your selection.

☒ Life sciences ☐ Behavioural & social sciences ☐ Ecological, evolutionary & environmental sciences

For a reference copy of the document with all sections, see nature.com/documents/nr-reporting-summary-flat.pdf

Life sciences study design

All studies must disclose on these points even when the disclosure is negative.

Sample size	In vitro blood clearance study: our in vitro experiment is a controlled engineering system where there is no variability of hemodynamic values (pressure and flow are maintained constant) in a vascular phantom that does not have changes in geometry. Therefore, each experiment performed in this controlled model is reproducible. No statistical analysis was performed in this study and reported in the manuscript.<br><br>Swine model neurovascular device assessments: no statistical analysis between the different imaging modalities in the swine model is present in the manuscript. We only reported inter-rater agreement between three expert image readers for making the diagnosis of malapposition or clot adherence to the surface of the device. Moreover, there is no feasible way to establish ground truth information, since it is well known that histological analysis of acutely implanted specimens would not be revealing. This is due to stent migration during sectioning and clot lysis or removal by sectioning since neither the device or clot are secured. Therefore, there is no statistically correct way to calculate the sample size required. Rather, we submit that with n = 8 replicate experiments giving n = 16 unique datasets is reasonable to show that there is excellent agreement when using HF-OCT to make the diagnosis amongst the radiologists as compared to CBCT and DSA.<br><br>Swine model tortuosity experiments: n = 8 animals were used for n = 16 assessments (i.e., bilateral brachial artery). All cases were successfully completed and imaging data sets characterized. There is no ground truth or control device available for a sample size calculation, since the device characterized in this study is the only intravascular imaging device capable of acquiring intravascular imaging data in elevated vascular tortuosity. No statistical analysis was performed in this study and reported in the manuscript. Experiment reproducibility was assessed in a total of n = 16 different swine brachial arteries from n = 8 different animals.<br><br>Cadaveric intracranial atherosclerosis: this is a qualitative comparison between HF-OCT and histology assessed by a pathologist (blinded to the HF-OCT data). No statistical analysis was performed in this study and reported in the manuscript.
Data exclusions	One partial dataset was excluded from HF-OCT image acquisition. During this case, the flow diverter was imaged. However, after the overlapping stent was implanted, the artery thrombosed and became excluded. As reported in the manuscript, n = 16 flow diverter complete HF-OCT datasets were acquired; however, n = 15 stent overlapping flow diverters were imaged (since HF-OCT cannot be performed in an occluded artery).
Replication	All replicate experiments (n = 16 tortuosity experiments, n = 16 flow diverter image acquisitions, and n = 15 stent overlapping flow diverter acqusitions) were successfully completed across three imaging modalities, with one data set excluded as noted above.
Randomization	In this study, we use in vitro, in vivo, and ex vivo modeling to assess HF-OCT as an imaging modality for neurovascular applications. The in vitro engineering system is controlled and reproducible. Imaging data using all three imaging modalities were acquired from all in vivo models. Similarly, for ex vivo models, data using both imaging and histopathology were obtained for all specimens. As such, no experiment requiring randomization was performed in this study.
Blinding	For the cadaveric study, the HF-OCT imaging reviewer was blinded to pathological assessment, and similarly the pathologist reading the histology was blinded to the results of HF-OCT. There was perfect agreement of plaque type between the reviewers. For the image assessment, 3 radiologists reviewed hundreds of images that were scrambled; meaning they were not able to associate the findings of any particular device (malapposition or clot formation) from one imaging modality to inform another.

Reporting for specific materials, systems and methods

We require information from authors about some types of materials, experimental systems and methods used in many studies. Here, indicate whether each material, system or method listed is relevant to your study. If you are not sure if a list item applies to your research, read the appropriate section before selecting a response.

Materials & experimental systems

n/a	Involved in the study	
☒	☐	Antibodies
☒	☐	Eukaryotic cell lines
☒	☐	Palaeontology
☐	☒	Animals and other organisms
☐	☒	Human research participants
☒	☐	Clinical data

Methods

n/a	Involved in the study	
☒	☐	ChIP-seq
☒	☐	Flow cytometry
☒	☐	MRI-based neuroimaging

Animals and other organisms

Policy information about studies involving animals; ARRIVE guidelines recommended for reporting animal research

Laboratory animals	Sus scrofa domesticus, 7 males, 1 female, 12-16 weeks old
Wild animals	Study did not involve wild animals.
Field-collected samples	Study did not involve samples collected from the field.
Ethics oversight	Institutional Animal Care and Use Committee, NIH

Note that full information on the approval of the study protocol must also be provided in the manuscript.

Human research participants

Policy information about studies involving human research participants

Population characteristics	Cadaveric specimens were obtained from three (3) patients older than 70 years of age with a history of smoking and coronary and/or peripheral artery disease
Recruitment	In this study, intracranial arterial specimens showing intracranial disease were selected to characterize the ability of HF-OCT to visualize plaque morphology vs. histopathology assessment. Patient selection does not have an impact on the results presented in this manuscript.
Ethics oversight	Per NIH guidelines and University of Massachusetts Medical School IRB guidance, specimens from deceased individuals are not human subjects research. Consent to use remains for medical research was provided by donors prior to being deceased.

Note that full information on the approval of the study protocol must also be provided in the manuscript.
